# Evaluation of the Effect of Anti-COVID-19 Mouthwashes on Shear Bond Strength of Composite Resin Restorations to Dentin and Enamel: An “In Vitro Study”

**DOI:** 10.1155/2022/3824796

**Published:** 2022-05-07

**Authors:** Farzaneh Shirani, Ali Mazdak, Peiman Mazaheri, Mehrangiz Shirani, Pouran Samimi

**Affiliations:** ^1^Department of Restorative Dentistry, Dental Materials Research Center, Dental Research Institute, School of Dentistry, Isfahan University of Medical Sciences, Isfahan, Iran; ^2^Dental Students Research Committee, School of Dentistry, Isfahan University of Medical Sciences, Isfahan, Iran; ^3^School of Dentistry, Isfahan University of Medical Sciences, Isfahan, Iran

## Abstract

**Objectives:**

Given the high prevalence of the coronavirus and the high risk of virus transfer to dentists, the use of mouthwashes, which can potentially eliminate this virus, is suggested before dental procedures. Since these mouthwashes may affect the bond strength of composite resin restorations to teeth, this study was conducted to investigate the effect of recommended mouthwashes on the shear bond strength of composite resin restorations to dentin and enamel in selective etch and rinse and two-step self-etch bonding systems.

**Methods:**

Five groups of posterior teeth (*n* = 15) were selected for five groups of cetylpyridinium chloride 0.07%, povidone-iodine 1%, hydrogen peroxide 1%, and chlorhexidine 0.2% as mouthwash and distilled water as the control group. The buccal enamel and lingual dentin of each tooth were rinsed after immersion in a mouthwash. After 20 seconds of enamel acid-etching and 15 seconds of dentin priming, they were impregnated with an adhesive, and composite cylinders were placed on the dentin and enamel surfaces of the tooth. The shear bond strength test was performed after 24 hours, and results were analyzed by ANOVA and paired *t*-test (*α* = 0.05).

**Results:**

The mean shear bond strength of enamel to composite was significantly (*p* < 0.05) higher than that of dentin to composite in each study group, but no significant difference was found between the mean shear bond strength of composite to enamel (*p* = 0.199) and to dentin (*p* = 0.335) after the use of mouthwashes and that of the control group.

**Conclusion:**

The use of mouthwashes used in this study did not have negative effects on the shear bond strength of composite to enamel and dentin.

## 1. Introduction

The rapid spread of coronavirus in a very short time in more than 100 countries indicates the incredibly high transmission capacity of this virus, which has caused concerns in the health systems of all countries [1]. Human-to-human coronavirus transmission occurs due to close contact with an infected person and exposure to cough, sneezing, respiratory droplets, or airborne contaminants [2].

Due to the large number of patients and the lack of effective treatments to treat COVID-19 so far, necessary measures have been taken to control the spread of the disease based on past epidemic experiences with similar viruses through hand hygiene, use of masks and mouthwashes, and social distancing [3].

Angiotensin-converting enzyme 2 (ACE2) has a great affinity to severe acute respiratory syndrome coronavirus (SARS-CoV) [4]. SARS-CoV-2 enters host cells through ACE2 receptors [5]. According to the latest information on COVID-19, since the number of ACE2 receptors in the epithelial cells of the salivary ducts is so high and even higher than the number of these receptors in the lung cells, asymptomatic patients have a high viral load in saliva [6]. Therefore, the healthcare providers, including dentists, who are in close contact with saliva are at high risk for the disease [7–9].

Antiseptic mouthwashes can kill the virus in the saliva, thereby reducing the chance of virus transmission through the mouth [6, 8]. Povidone-iodine (PVP-I) [10–15], cetylpyridinium chloride (CPC) [13, 14, 16], hydrogen peroxide [10, 13, 15], and chlorhexidine (CHX) [10, 15] are mouthwashes that kill enveloped viruses such as coronavirus.

PVP-I is composed of iodine and a water-soluble polymer called polyvinylpyrrolidone (PVP). PVP-I possesses antimicrobial properties through the release of iodine. Iodine penetrates into microbes, oxidizes nucleic acids, and breaks down the proteins. Thus, PVP-I impairs the virus through damage to the membrane and multiple metabolic pathways [17]. Gargling and rinsing the mouth with solutions containing 1% PVP-I for 30 seconds have shown more than 99.99% of antiviral properties against coronavirus [12, 18].

Cetylpyridinium chloride (CPC) or N-hexadecyl pyridinium chloride is a water-soluble quaternary ammonium compound that is highly cationic at neutral pH [19]. CPC is likely to inactivate the virus through capsid destruction as well as lysosomotropic activity, which is common for tetravalent ammonium compounds [20]. Vinet and Zhedanov reported that mouthwashes containing 0.07% CPC have very high anti-SARS-CoV-2 properties [21]. The recommended time for the use of this mouthwash is 30-60 seconds [22]. The American Dental Health Association (ADHA) has recommended the use of mouthwashes containing CPC against COVID-19 before dental procedures [23].

Hydrogen peroxide (H2O2) is a widely used chemical and antimicrobial compound that has been shown to work against several human viruses, the most susceptible of which are the coronavirus and influenza [24]. By releasing oxygen free radicals, H2O2 targets the lipid envelope of these viruses, especially SARS-CoV-2 [13]. The use of mouthwashes containing 1% hydrogen peroxide for 30-60 seconds has been suggested to reduce the coronavirus load in saliva [22, 25].

Chlorhexidine (CHX) is a cationic bisbiguanide that disintegrates the cytoplasmic membrane of microorganisms by disrupting their cytoplasmic balance, causing the sedimentation of cell content [26]. Chlorhexidine is one of the most common disinfectants prescribed in dentistry [27]. For example, use of CHX reduces Porphyromonas gingivalis load and patient's inflammatory response around dental implants [28] and also reduces periodontitis and peri-implantitis and peri-implant crestal bone loss [29]. An in vitro study on the effect of 0.12% chlorhexidine mouthwash on viruses has shown that this concentration can reduce the coated viruses [13]. The recommended time for the use of this mouthwash is 30-60 seconds [22]. The American Dental Health Association (ADHA) has recommended the use of mouthwashes containing CHX against COVID-19 before dental procedures [23].

Nowadays, due to the greater tendency of patients to use tooth-colored restorations, greater protection of dental tissue, and many improvements in the mechanical properties of composite resin materials, the use of these materials has increased, turning them into one of the most widely used materials in restorative dentistry [30]. For the success of composite resin restorations, it is necessary to establish a durable bond between these materials and the tooth structure [31]. The confounding factors can also affect the tooth-composite quality and bond [32].

Studies investigating the effect of chlorhexidine as a cavity disinfectant have evaluated different concentrations of this substance ranging from 0.002 to 5%. Most of these studies have reported positive effects of this material on the bond strength of dentin to composite resin restorations. It seems that chlorhexidine has the ability to dissolve the smear debris and also to increase the dentin's surface energy, which improves the wettability of primers. On the other hand, several studies have shown that chlorhexidine has a negative effect on the bond strength of dentin bonding systems [33].

Silva et al. investigated the effect of 2% iodine solution (iodine disclosing/disinfecting solution (i2dds) 2%) on the bonding strength of a composite for 20 seconds and reported a decrease in this bond strength. Suma et al. also examined the effect of 0.3% potassium iodide solution on the dentin bond strength for 60 seconds and reported a decrease in the bond strength [34, 35].

Ercan et al. evaluated the use of 3% hydrogen peroxide for 20 seconds when using etch and rinse and self-etching systems. They reported that the composite-dentin bond strength decreased while using the self-etching systems [36]. Reddy et al. examined two self-etching systems after exposing the dentin to 3% hydrogen peroxide for 20 seconds and reported a decrease in bond strength in both systems [37]. Bond strength reduction in H2O2 treated tooth can be caused by remaining H2O2 in the collagen matrix that broke down into water and oxygen. Releasing oxygen can interfere with infiltration of resin into etched dentin. Also, it can inhibit resin polymerization [38].

Among the quaternary ammonium compounds, Sharma et al. investigated the effect of using 1% benzalkonium chloride for 15 seconds on the dentin-composite bond strength 24 hours and 12 months after application of the adhesive. Their results showed decreased bond strength only in the 12-month period [39].

When a patient visits a dentist, due to their close contact, it is recommended to use one of the common mouthwashes immediately before starting the dental procedure to reduce the risk of disease transmission. On the other hand, there is a possibility of the effect of these materials on the dentin and enamel properties and subsequently on the bond strength of resin composites. Therefore, this study evaluated the effect of four types of mouthwash, including 0.07% cetylpyridinium chloride, 1% povidone-iodine, 1% hydrogen peroxide, and 0.2% chlorhexidine, on the shear bond strength of composite resin materials to enamel and dentin. The null hypothesis of this study was that the use of these mouthwashes would not affect the bond strength of composite to dentin and enamel in selective etch and rinse and two-step self-etch bonding systems.

## 2. Materials and Methods

### 2.1. Selection of Teeth for Analysis and Preparation

**Figure 1:**
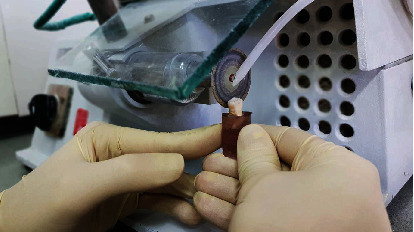
Dentin exposure procedure.

Seventy-five noncarious posterior teeth (molars and premolars) extracted for periodontal and orthodontic treatments were selected. The ISO/TS 11405: 2015 [40] gives guidance on selecting substrates and mentions that using premolars and molars is ideal. The teeth were kept in 0.2% thymol (thymol: Applichem GmbH, Ottweg 4, D-64291 Darmstadt, Germany) solution since extraction. The teeth were divided into 5 groups (A to E). Each group consisted of 5 premolars and 10 molars that were matched between the groups in terms of dimensions. After classification and before the start of the procedure, each tooth was mounted in a three-component epoxy resin (Aron Polymer, Tehran, Iran) in the form of a cylinder with a diameter of 3 cm. The buccal surface of the tooth was considered to measure the enamel-composite bond, and the lingual surface of the same tooth was considered to measure the dentin-composite bond. Using the diamond disc of a cutting machine for animal samples (Vafaie Industrial Co., Tehran, Iran), the enamel of the lingual surface of the tooth was prepared under cooling water, and the dentin was exposed (Figure 1) and polished with a 600-grit sandpaper for 30 seconds.

### 2.2. Immersion in Mouthwash Procedure

Each tooth was immersed in its group mouthwash according to the following instructions before the bonding procedure as follows:

Group A was placed in 15 ml 0.07% cetylpyridinium chloride mouthwash for 1 minute.

Group B was placed in 15 ml 1% povidone-iodine mouthwash for 30 seconds.

Group C was placed in 15 ml 1% hydrogen peroxide mouthwash for 1 minute.

Group D was placed in 15 ml 0.2% chlorhexidine mouthwash for 1 minute.

Group E, as a control group, was placed in 15 ml distilled water for 1 minute.

All teeth were rinsed under strong water flow for 20 seconds after being removed from the mouthwash.

### 2.3. Bonding Procedure

After immersing in the mouthwash and rinsing and before the bonding procedure, the buccal enamel surface was removed under cooling water using the diamond disc of a cutting machine for animal samples (Vafaie Industrial Co., Tehran, Iran) to perform the simulation with beveling stage in composite cavities.

#### 2.3.1. Dentin Bonding Procedure

After drying the teeth on the lingual surface, Clearfil Liner Bond F Primer (Kuraray Noritake Dental Inc., 1621 Sakuzu, Kurashiki, Okayama, Japan) was scrubbed on the dentin surface by a disposable micro applicator (Woodpecker, China) for 15 seconds. Then, it was dried for 15 seconds to remove the solvent with oil-free compressed air. The Clearfil Liner Bond F (Kuraray Noritake Dental Inc., 1621 Sakuzu, Kurashiki, Okayama, Japan) was then applied to the dentin surface by a disposable micro applicator (Woodpecker, China). It was then thinned with oil-free compressed air at a distance of 5 cm and cured by DemiPlus light curing device (Kerr, USA) with a light intensity of 1330-1100 mw/cm and a wavelength of 470-450 nm for 20 seconds from the minimum distance. Then, composite cylinders (Filtek™ Supreme Ultra Universal Restorative, 3M, USA) with a diameter of 2.5 mm and a height of 2 mm were placed on the dentin surface and cured by the DemiPlus light curing device (Kerr, USA) with a light intensity of 1330-1100 mw/cm and a wavelength of 470-450 nm once from the mesial side of the cylinder for 20 seconds and once from the distal side of the cylinder for 20 seconds.

#### 2.3.2. Enamel Bonding Procedure

**Figure 2:**
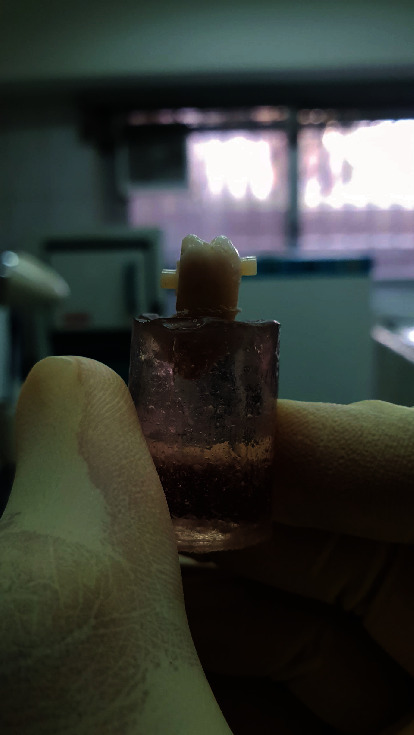
. A prepared sample.

After drying the tooth, the enamel was etched with Ultra-Etch 35% phosphoric acid gel (Ultradent Products, Inc., USA) for 20 seconds. It was then rinsed with water for 20 seconds, with a water-air mixture for 20 seconds and with air for 20 seconds. The Clearfil Liner Bond F was then scrubbed on the enamel surface by a disposable micro applicator (Woodpecker, China) and thinned with oil-free compressed air from a distance of 5 cm. It was then cured with a DemiPlus light curing machine (Kerr, USA) with a light intensity of 1330-1100 mw/cm and a wavelength of 470-450 nm for 20 seconds from the minimum distance. Next, composite cylinders with a diameter of 2.5 mm and a height of 2 mm were placed on the enamel surface and cured by the DemiPlus light curing device (Kerr, USA) with a light intensity of 1330-1100 mw/cm and a wavelength of 470-450 nm once from the mesial side and once from the distal side of the cylinder, each time for 20 seconds (Figure 2).

### 2.4. Storage and Shear Bond Strength Test

**Figure 3:**
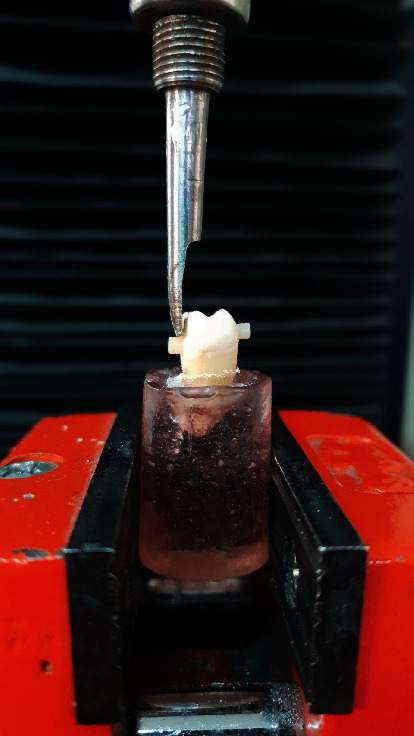
Sample's position on the universal testing machine and the blade's position respect to the sample.

Following the above steps, each tooth was placed in 37°C distilled water for 24 hours in a digital incubator (Behdad, Tehran, Iran, 01154). Then, the shear bond strength of each sample was measured by an electromechanical universal testing machine (K-21046, Walter+Bai, Switzerland) as follows:

The tooth sample was fixed inside the clamp of the device from the mounted site. The blade was then placed perpendicular to the longitudinal axis of the crown at the bonding site of composite to dentin and enamel (Figure 3). Next, a force of 0.5 mm/min [30] was applied until the composite cylinders were separated from the tooth surface. The amount of force required to separate the composite cylinders was determined and calculated in MPa by dividing it by the bonding surface of the shear bond strength.

The fracture mode of each sample was described after examination under a light microscope as follows:

(1) If the composite was isolated from the tooth surface from the bonding site, the fracture mode was reported as adhesive
(2) If the enamel or dentin was fractured under the test, the fracture mode was reported as enamel/dentin cohesive
(3) If the composite was fractured under the test, the fracture mode was reported as composite cohesive
(4) If part of the tooth or composite was fractured when the composite was detached from the bonding site, the fracture mode was reported as mixed, which itself is of two types:
  - Fracture of a part of dentin or enamel with the detachment of the bond, which is called dentin/enamel mixed cohesive
  - Fracture of a part of the composite with the detachment of the bond, which is called composite mixed cohesive

The results of fracture strength and fracture mode were analyzed by ANOVA, paired *t*-test, and chi-square test. The significance level was set at *α* = 0.05.

The procedures are summarized in Table 1.

## 3. Results

First, the normality of the research data was examined and confirmed using the Kolmogorov-Smirnov test (*p* > 0.05). The results of the bond strength of different study groups are given in Table 2.

While the highest dentinal bond strength belonged to the chlorhexidine mouthwash group and the lowest to the control group, the mean bond strength of dentin to the composite resin impregnated with different types of mouthwashes in different groups was not significantly different from that of the control group (*p* = 0.335). The highest enamel bond strength belonged to cetylpyridinium chloride mouthwash, and the lowest amount belonged to hydrogen peroxide, but the bond strength of composite resin to enamel impregnated with different types of mouthwashes was not significantly different in different groups compared to the control group (*p* = 0.199).

**Figure 4:**
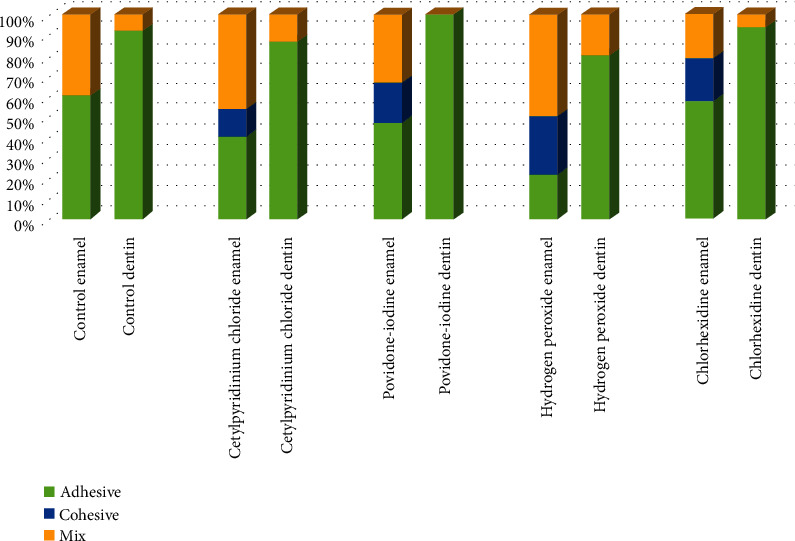
Comparison of enamel and dentin fracture modes in five study groups.

As shown in Figure 4, the highest fracture mode in dentin bonding was of adhesive type, but there was no significant difference between the groups (*p* = 0.141). As for the enamel, three types of fracture, including adhesive, cohesive, and mixed, were observed, but no significant difference was observed between the groups (*p* = 0.442).

## 4. Discussion

The results of this study confirmed the null hypothesis of the study, indicating that the use of mouthwashes immediately before the start of the restoration procedure did not have a negative effect on the bond strength of composite to dentin and enamel in two-step self-etch bonding systems using selective enamel etching. The selective enamel etching system was used for bonding the enamel and dentin because mild self-etches cannot dissolve the enamel hydroxyapatite crystals, which are larger, denser, and more consistent than the dentin hydroxyapatite crystals, create a deep micromechanical gear, and achieve a stable enamel bonding [41].

Therefore, self-etching adhesives should be combined with selective enamel etching with phosphoric acid. In this system, the advantages of selective enamel etching and a bonding agent without additional compounds and solvents can be used to achieve long-term stable results. Further, using an acidic primer without acid contamination, the overdemineralization of dentin and postrepair sensitivity can be prevented at the dentin level [41]. Today, phosphoric acid does not have a desirable effect on dentin because 3 to 6 microns of dentin surface are completely demineralized, and a network of collagen remains, into which the resin hardly penetrates and is hybridized. Incomplete penetration of resin into the exposed collagen network creates a thick hybrid layer without minerals, which has less strength and is prone to hydrolytic and enzymatic degradation [41].

The adhesive used in this study is a mild self-etching system with a long history of research and is considered the gold standard in dentin bonding systems [41, 42]. In this study, the immediate bond strength of the composite to enamel and dentin was evaluated, and it seems that applying thermal cycles and evaluating the bond strength after aging can provide bigger achievements in this field. Moreover, dentin and superficial enamel were evaluated because mouthwashes were used by the patient before any dental procedure. However, because enamel and dentin are permeable, mouthwashes with known and unknown properties may affect the bond strength of composites.

Assessing the bond strength of the surface enamel is highly important because the strength and durability of the enamel bonding play an important role in the success of dental restorations, such as direct restorative procedures in various composite cavities. For all restorative dental procedures, it has always been tried to maintain maximum enamel bonding. On the other hand, dentinal surface bonding was also evaluated in this study because many patients have exposed dentin in their mouth following gingival resorption and cementum loss, which can be a substrate for composite resin in lesions such as noncarious cervical lesions (NCCLs) [43]. Moreover, following the loss of enamel due to decay, erosion, and abrasion, we may still encounter a dentinal surface substrate in the mouth, which may be followed by composite bonding in the treatment plans. In addition, after rinsing the mouth and even after preparing the cavity, the effects left by mouthwashes may still lead to weaker bond results in the composite.

To evaluate the bond strength, the enamel and dentin of one tooth were used to compare dentin and enamel. The results indicated that the immediate bond strength of composite to enamel was significantly higher than that of composite to dentin using the bonding system used in this study, which is consistent with the results of many studies.

The compounds used as mouthwashes in this study have been investigated in other studies as a cavity cleanser or an antibacterial agent or inhibitor of matrix metalloproteinases (MMPs). However, due to different uses and applications of these compounds for various purposes, different results have been obtained.

In a systematic review, Coelho et al. [33] reported that the use of chlorhexidine (regardless of the concentration used) before the application of the adhesive system did not change the values of dentinal bond strength but increased these values in some of them. Moreover, in this review, the majority of studies that used the 2% concentration showed this concentration had positive effects on the bond strength of composite resin restorations to dentin. Furthermore, in this systematic review, Sharma et al. [39] showed that using 2% chlorhexidine before the self-etching system reduced the bond strength, but other studies reported an increase in the bonding strength to dentin when the chlorhexidine concentration was decreased to 1% [33].

Although in dentinal bonding the chlorhexidine mouthwash group showed the highest bond strength compared to other groups, the present study showed that using 0.2% chlorhexidine mouthwash for 60 seconds (after 24 hours) had no significant effect on the shear bond strength of composite to dentin and enamel. Differences between the results of this study and those of studies that have indicated the positive effect of chlorhexidine on the bond strength of the resin composite can be related to different dentin sections, different bonding systems and protocols, different chlorhexidine concentrations, and different application times.

Chlorhexidine has been used to inhibit MMP2 in all previous studies after cavity preparation and acid etching in etch and rinse bonding systems [44], while in the present study, it was used as a mouthwash before cavity preparation. Chlorhexidine has been shown to have more positive effect on the bond durability of composite to dentin in etch and rinse bonding systems [45, 46], while the present study evaluated the immediate bond strength and the two-stage self-etch bonding system without the contact of dentin with acid. Chlorhexidine mouthwash causes tooth discoloration after long-term application [27, 47], but according to the results obtained in this field, its short-term application for microbial detoxification does not have adverse effects on the bond strength of resin to enamel and dentin, and it can be used without any concerns.

Suma et al. and Da Silva et al. investigated the effect of disinfectants containing iodine on the shear bond strength of composite to dentin using self-etching systems. Suma et al. examined the effect of this material at a concentration of 0.3% for 60 seconds (after one week), and Da Silva et al. examined the effect of this substance at a concentration of 2% for 20 seconds (after 24 hours). Both of these studies revealed that disinfectants containing iodine reduced the composite-dentin bond strength [34, 35]. However, this result was not obtained in the present study, which can be due to different dentin sections, different application times, different bonding systems, different bonding protocols, and different iodine concentrations.

Ercan et al. evaluated 3% hydrogen peroxide for 20 seconds when using etch and rinse and self-etching systems and reported that the bond strength of the composite to dentin was reduced when using the self-etching systems [36]. Reddy et al. examined two self-etching systems after exposing the dentin to 3% hydrogen peroxide for 20 seconds and reported a decrease in bond strength in both systems [37]. In the present study, 1% hydrogen peroxide mouthwash was used for 60 seconds, but no significant change was observed in the composite-dentin bond strength. This difference between these two studies can indicate the effect of higher concentrations of hydrogen peroxide on the bond strength reduction.

Among the quaternary ammonium compounds, Sharma et al. investigated the effect of 1% benzalkonium chloride on dentinal bond strength for 15 seconds, 24 hours, and 12 months after application of the adhesive. In this study, decreased bonding strength was reported only in the 12-month period [39]. The present study investigated the effect of another quaternary ammonium compound, 0.07% cetylpyridinium chloride, on the bond strength of the composite to enamel and dentin for 60 seconds and showed no significant change compared to the control group. Due to the lack of studies on the effect of this material on the bond strength of composite restorations, further studies are needed in this area. It should be noted that cetylpyridinium chloride with antimicrobial effects was also added to the bonding agents [41].

Unlike other studies mentioned in this study, the present study evaluated the effect of mouthwashes on the bond strength of composite restorations to enamel and indicated no significant change in any of the groups compared to the control group. Due to the lack of studies on the effect of different mouthwashes on the bond strength of composite restorations to enamel, further studies are needed to shed more light on this domain of research.

In any case, the collection of study samples (noncarious human teeth) was really hard. Therefore, further studies with more sample sizes are needed.

## 5. Conclusions

Within the limitations of this in vitro study, the use of 0.07% cetylpyridinium chloride mouthwash for 1 minute, 1% povidone-iodine mouthwash for 30 seconds, 1% hydrogen peroxide mouthwash for 1 minute, and 0.2% chlorhexidine mouthwash for 1 minute, before dental procedures, did not have negative effects on the shear bond strength of resin composite restorations to enamel and dentin in selective etch and rinse and two-step self-etch bonding systems.

## Figures and Tables

**Table 1:** . Summary of the procedures.

Group		Procedure steps
A	Dentin exposure Immersion in 15 ml 0.07% cetylpyridinium chloride mouthwash for 1 minute	Drying the tooth, scrubbing the primer on the dentin surface for 15 seconds and removing the solvent, placing the bond on the dentin surface and thinning and curing it for 20 seconds, placing the resin composite cylinder on the dentin, and curing it for 20 seconds from the mesial and distal side, respectively
B	Dentin exposure Immersion in 15 ml 1% povidone-iodine mouthwash for 30 seconds	
C	Dentin exposure Immersion in 15 ml 1% hydrogen peroxide mouthwash for 1 minute	Removing the buccal surface enamel, drying the tooth, etching the enamel surface with phosphoric acid for 20 seconds, rinsing with water for 20 seconds, rinsing with water and air mixture for 20 seconds, drying with air for 20 seconds, scrubbing the bond on the enamel surface, thinning and curing it for 20 seconds, placing the resin composite cylinder on the enamel, and curing it for 20 seconds from the mesial and distal side, respectively
D	Dentin exposure Immersion in 15 ml 0.2% chlorhexidine mouthwash for 1 minute	
E	Dentin exposure Immersion in 15 ml distilled water for 1 minute	

**Table 2:** Fracture strength values in different study groups.

Group	Substrate	Mean (MPa)	Std. deviation	Std. error mean	*t*	df	Sig.	Upper bond (MPa)	Lower bond (MPa)
Cetylpyridinium chloride	Enamel	33.4460^a^	6.62433	1.71039	6.332	14	.000	37.1144	29.7776
	Dentin	21.1853^b^	7.06013	1.82292				25.0951	17.2756
Povidone-iodine	Enamel	32.9620^a^	7.09081	1.83084	5.254	14	.000	36.8888	29.0352
	Dentin	18.7427^b^	5.39910	1.39404				21.7326	15.7527
Hydrogen peroxide	Enamel	27.9007^a^	7.04100	1.88179	3.152	13	.008	31.9661	23.8354
	Dentin	19.1600^b^	8.29973	2.28719				23.7562	14.5638
Chlorhexidine	Enamel	30.6114^a^	7.41184	1.98090	2.829	13	.014	34.8909	26.3320
	Dentin	22.8467^b^	7.83380	2.16950				27.1849	18.5085
Control	Enamel	32.0540^a^	5.78642	1.25605	4.805	12	.000	35.2584	28.8496
	Dentin	17.9108^b^	6.35785	1.76335				21.7528	14.0688

^∗^Numerical values with dissimilar power are significantly different from each other.

## Data Availability

The data that support the findings of this study are available from the corresponding author, upon reasonable request.
